# MASQC: Next Generation Sequencing Assists Third Generation Sequencing for Quality Control in N6-Methyladenine DNA Identification

**DOI:** 10.3389/fgene.2020.00269

**Published:** 2020-03-24

**Authors:** Siqian Yang, Yaoxin Wang, Ying Chen, Qi Dai

**Affiliations:** ^1^College of Life Sciences and Medicine, Zhejiang Sci-Tech University, Hangzhou, China; ^2^State Key Laboratory of Ophthalmology, Zhongshan Ophthalmic Center, Sun Yat-sen University, Guangzhou, China

**Keywords:** DNA N6-methyladenine, MeDIP-seq, SMRT-seq, eukaryotes, prokaryotes

## Abstract

DNA N6-methyladenine (6mA) modification has been discovered as the most prevalent DNA modification in prokaryotes and eukaryotes, involving gene expression, DNA replication and repair, and host-pathogen interactions. Single-molecule real-time sequencing (SMRT-seq) can detect 6mA events in prokaryotic and eukaryotic genomes at the single-nucleotide level. However, there are no strict and economical quality control methods for high false-positive 6mA events in eukaryotic genomes. Therefore, by analyzing the distribution of 6mA in eukaryotic and prokaryotes, we proposed a method named MASQC (MeDIP-seq assists SMRT-seq for quality control in 6mA identification), which can identify 6mA events without doing the whole genome amplification (WGA) sequencing. The proposed MASQC method was assessed on two eukaryotic genomes and six bacterial genomes, our results demonstrate that MASQC performs well in quality control of false positive 6mA identification for both eukaryotic and prokaryotic genomes.

## Introduction

Epigenetics is a study based on changes in gene expression levels caused by non-gene sequence changes. The epigenetic control of gene expression mainly includes DNA methylation, histone modification, chromosomal remodeling and non-coding RNA regulation (Geiman and Robertson, 2002), among which DNA methylation modification plays an important role in the regulation of gene expression in epigenetics (Calicchio et al., 2014). It is well known that C5-methylcytosine (5mC) and N6-methyldeoxyadenosine (6mA) are the most abundant and predominant DNA methylation modifications and play a crucial role in both eukaryotic and prokaryotic life processes (Ratel et al., 2006; Liu et al., 2016).

The 5mC modification has been well-studied in prokaryotes and eukaryotes which regulates diverse biological functions and life processes. In contrast, the 6mA modification commonly associates with restriction modification (RM) systems that defend hosts against invading foreign genomes (Fu et al., 2015), while the in-depth research on it has not made significant progress due to the limitation of previous detection technology. Subsequently, the development of specific antibodies and Next Generation Sequencing technology brought a glimmer of light to this problem, which could detect the conservative regions 6mA events occur in. Based on these techniques, previous researches have been reported the detection of 6mA events in *C. elegans* (Greer et al., 2015), *D. melanogaster* (Zhang et al., 2015), *Homo sapiens* (Xiao et al., 2018), *S. cerevisiae* (Mondo et al., 2017), and *Chlamydomonas reinhardtii* (Fu et al., 2015).

At present, a variety of methods have been proposed to detect the 6mA events in eukaryotic and prokaryotic genomes, including bisulfite sequencing (Svadbina et al., 2004), methylated DNA immunoprecipitation sequencing (MeDIP-seq) (Zhao et al., 2014), restriction enzyme-based 6mA sequencing (RE-seq) (Luo et al., 2016), single-molecule real-time sequencing (SMRT-seq) (Flusberg et al., 2010) and Nanopore sequencing (ONT-seq) (Branton et al., 2008). Previously, the whole genome DNA methylation detection mainly relied on bisulfite sequencing or the next generation sequencing of methylated DNA immunoprecipitation (Shanmuganathan et al., 2013), but it was difficult to accurately identify the methylation of genomic repeat regions due to the short reads. Although methylated DNA immunoprecipitation (MeDIP) can detect the region of the 6mA event on the genome, it is not possible to identify the 6mA event on a single nucleotide (Zhu et al., 2016; Rand et al., 2017).

Single-molecule real-time (SMRT) sequencing by Pacific Biosciences enables the genome-wide mapping of 6mA modification at single nucleotide resolution and even single molecule level by monitoring pulsed fluorescence of single nucleotide events (Koren and Phillippy, 2015; VanBuren et al., 2015). The time at which SMRT sequencing monitors the pulsed fluorescence of a single nucleotide is termed as inter-pulse duration (IPD) (Flusberg et al., 2010; Feng et al., 2013). The IPD ratio is derive from that ratio of the IPD observed from the reference location on each strand and the control IPD. Control IPDs are supplied by either an *in silico* computational model or observed IPDs from unmodified “control” DNA. IPD ratio reflects the deviation of IPDs distribution from the expected level, and the IPD deviations are highly related to neighboring nucleotides modifications. With the help of the IPD ratio from SMRT sequencing, a host of 6mA events have been detected in hundreds of bacterial and archaeal genomes (Sanchez-Romero et al., 2015; Blow et al., 2016). Although SMRT sequencing has also been used to detect 6mA events in eukaryotes (Greer et al., 2015), its application still faces enormous challenges.

There are many differences among the 6mA events in eukaryotic and prokaryotic organisms. Firstly, the 6mA abundance (6mA/A) in eukaryotes is lower than that in prokaryotes (Casadesus and Low, 2006), and the detection of DNA methylation modification has a certain of false positive rate (FPR). In eukaryotes, the lower the 6mA abundance, the higher the 6mA FPR, the true 6mA events will be overwhelmed by a large number of false positive events (Fang et al., 2012). Secondly, 6mA events in prokaryotes are highly sequence specificity due to participation in the RM system. Typically, 6mA events in the prokaryotic genome occur almost (>95%) on several particular motifs. In contrast, 6mA events are motif driven weakly in eukaryotes, probably resulting from participation in functional regulation rather than the RM system (Wu et al., 2016). For instance, a small fraction (<3%) of occurrences on motifs have been recognized as true 6mA events in *C. reinhardtii* and *C. elegans*. Lastly, other types of DNA modifications (DNA damage, 5mC and derivatives produced during demethylation) in adjacent bases may interfere with the IPD ratio of adenine sites, leading to high FPR in the 6mA events detection. In order to reduce the FPR, the whole genome amplified DNA (WGA DNA, unmethylated DNA) was required to do sequencing as a control, but the WGA SMRT sequencing is extremely expensive. There is a pressing need to develop an efficient cost-effective computational method to reduce the FPR of 6mA events identification.

With the above problems in mind, we proposed a statistical method to control the FPR of 6mA events identification with the help of MeDIP-seq datasets. Take full advantage of the peak regions from MeDIP-seq datasets, we identified the 6mA events detected by SMRT sequencing and calculated a threshold of IPD ratio directly to filter out a large number of false positive events. Besides, the proposed method makes no use of WGA data, which significantly lowers the cost of sequencing.

## Materials and Methods

### MeDIP Sequencing Data and SMRT Sequencing Data

The raw data files of SMRT-seq and MeDIP-seq used in this study were downloaded from NCBI SRA database, including MeDIP-seq raw data for *C. elegans* (Greer et al., 2015), SMRT-seq dataset for *C. elegans* from Shi, Y.’s paper result (Greer et al., 2015), MeDIP-seq raw data for *C. reinhardtii* (Fu et al., 2015), SMRT-seq raw data and WGA raw data for *C. reinhardtii* (Zhu et al., 2018), MeDIP-seq raw data and SMRT-seq raw data for six bacterial genomes (*E. coli*, *B. subtilis*, *E. faecalis*, *S. aureus*, and *S. enterica*) (McIntyre et al., 2019). The detail description of these raw data can be found in Supplementary Material.

### MeDIP-seq Assists SMRT-seq for 6mA Quality Control (MASQC) Framework

**FIGURE 1:**
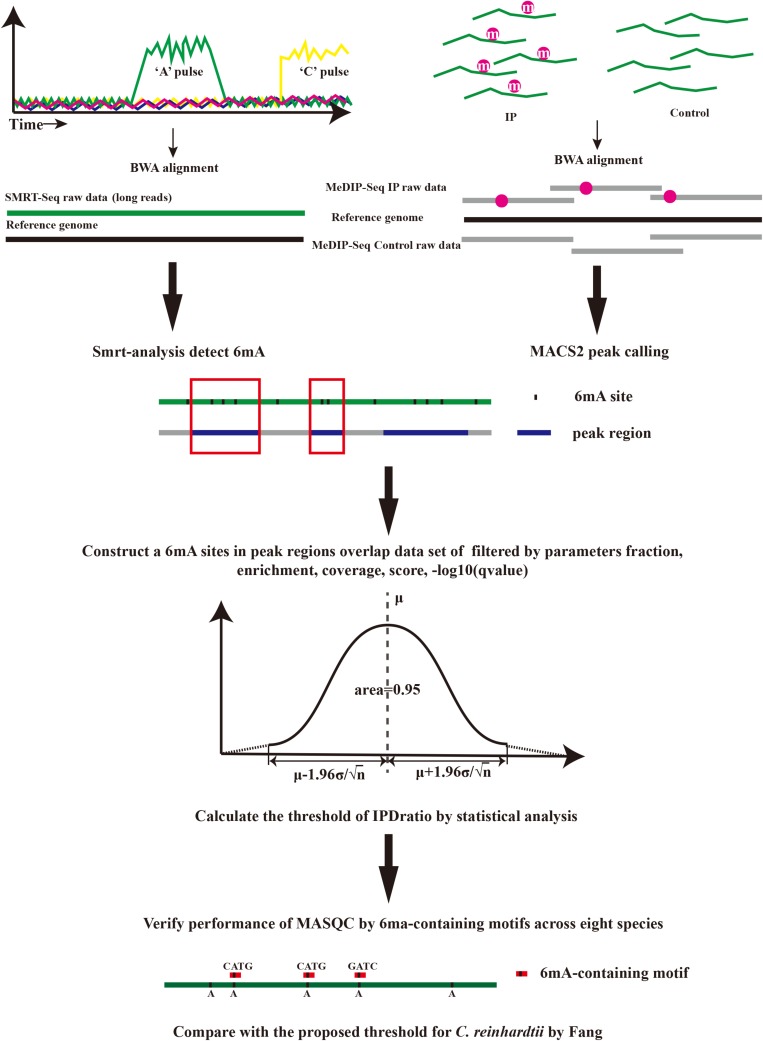
The overview of MASQC method.

MASQC is a proposed statistical method that combines MeDIP-seq with SMRT-seq. In MASQC, the input files include a reference genome, h5 format files generated by PacBio RSII sequencers and MeDIP-seq data generated by Illumina sequencers, the output results include 6mA peaks regions files and datasets of 6mA sites before and after threshold filtering. MASQC contains several steps shown in Figure 1.

(1) The input MeDIP-seq datasets consist of a reference genome, Input and IP reads files. Input and IP reads were aligned to their reference genomes using BWA-MEM (Li and Durbin, 2009), and the peaks were called by using MACS2 (–nomodel) (Zhang et al., 2008). Peak regions were in output file which is end with “narrow. Peak.”
(2) PacBio SMRT Tools (version 2.3.0) was used to detect DNA 6mA modifications for each strain^1^. In brief, an initial filtering step removes reads containing adapters, short reads and the other low quality reads with cutoffs (MapQV ≤ 240, read quality ≤ 0.75, read length ≤ 500 nt, and subread length ≤ 50 nt) in Eukaryotes (Liang et al., 2018), but using default parameters in prokaryotes. The detailed analysis workflow is as follows: Firstly, the clean reads were aligned to the corresponding reference genome of each strain by pbalign. Secondly, the polymerase kinetics information was loaded after being processed by loadChemistry. py and loadPulses. Finally, the post-aligned datasets were sorted by using cmph5tools and the 6mA was identified by using ipdSummary. py script. 6mA events with less than 50-fold coverage per chromosome of each strain were excluded for further analysis to ensure reliable detection.
(3) MASQC uses peak regions to construct a new conservative dataset of 6mA events in overlap regions which contains key features in both MeDIP-seq and SMRT-seq. These several features are extracted from the output modification files and peak files, including coverage, fraction, score, Enrichment and −10 log (*q*-value) that are described as below.

(i) Coverage refers to the default coverage of the position which has a 6mA base, coverage at that position is at least 10x.
(ii) Fraction refers to the fraction of reads aligning to this position which has a 6mA base.
(iii) Score refers to the reliability of 6mA come from SMRT analysis, 20 is the minimum default threshold for the datasets, and corresponds to a *p*-value of 0.01. Score of 30 corresponds to a *p*-value of 0.001.
(iv) Enrichment refers to the enrichment factor of peak (relative to random Poisson distribution with local lambda).
(v) −10 log (*q*-value) evaluates the reliability of this peak [default *q*-value < 0.05 correspond to −10 log (*q*-value) > 1.3, and *q*-value < 0.01 correspond to −10 log (*q*-value) > 2].

IPD ratio is not stable because it can be influenced by various factors (background value, noise, etc.), but the peak regions of MeDIP-seq are conservative and reliable so the peak-filtered sites are more reliable. We calculated the mean of these reliable IPD ratios and got the confidence interval of the mean to filter the most reliable sites from the raw data. Combined with the SMRT sequencing and MeDIP-seq principle analysis, the higher the probability of 6mA methylation events in the peak regions, the higher the detected fraction of 6mA abundance (0.7∼1). For the sake of obtaining the closest fully true dataset, MASQC firstly performs stricter filtering on the peak regions [enrichment ≥ 1, −10 log (*q*-value) ≥ 2] and the sites detected by SMRT analysis (coverage ≥ 50, score ≥ 30, fraction ≥ 0.7). The filtered dataset has been exceedingly close to the expected fully true dataset. We hold that the expected fully true dataset distribution follows a normal distribution, consequently the sample is extracted from the filtered dataset, and the overall distribution is verified by the sample distribution. The normal distribution equation is

(1) $$f\,\left(x\right)=\frac{1}{\sqrt{2\,\pi }\,\sigma }\,\exp\,\left(-\frac{{\left(x-\mu \right)}^{2}}{2\,\sigma^{2}}\right)$$

where μ is the mean of sample, σ is the standard deviation of sample. If a random variable X obeys a normal distribution with μ and variance σ^2^, it is defined as N (μ, σ^2^). The equation indicates that μ of the normal distribution determines the position, and its standard deviation σ determines the magnitude of distribution. When μ = 0 and σ = 1, the normal distribution is the standard normal distribution. According to the central limit theorem, the mean and variance of the population can be calculated based on the sample. Therefore, the 95% confidence interval of the overall IPD ratio can be inferred from the mean of the sample IPD ratio. MASQC obtains the 95% confidence interval by Student’s test. When the variance σ^2^ of population X is unknown, the variance *S*^2^ of sample is instead of σ^2^, so the 95% confidence interval of μ is

(2) $$\left[\bar{X}-\frac{S}{\sqrt{n}}\,t_{\frac{\alpha }{2}}\,\left(n-1\right),\bar{X}+\frac{S}{\sqrt{n}}\,t_{\frac{\alpha }{2}}\,\left(n-1\right)\right]$$

Where α = 0.05, $t_{\frac{\alpha }{2}}\,\left(n-1\right)=1.96$ are according to the T-distribution table, the number of sample *n* is 30. MASQC takes $\bar{X}-\frac{S}{\sqrt{n}}\,t_{\frac{\alpha }{2}}\,\left(n-1\right)$ the lower bound of the confidence interval as a threshold.

(4) Given the threshold of IPD ratio, most of false positive detection of 6mA events can be filtered out by threshold.

(3) $$T=N_{i\geq \mathrm{thres}}$$

Where *T* denotes the 6mA events after quality control, *N* denotes the total 6mA events and *i* denotes the threshold of IPD ratio.

### Evaluation and Verification

We compared the number of published 6mA-containing motifs for each species before and after threshold filtering got from MASQC. Three tests were used to evaluate the performance of MASQC. We also analyzed the change of the proportion of published 6mA-containing motifs in peak regions before and after threshold filtering to verify MASQC. *P*_1_, *P*_2_, *P*_3_, and *P*_4_ denote the proportions of the single motif in states PacBio, PacBio + MeDIP, PacBio + threshold and PacBio + MeDIP + threshold.*I* and *D* are the increase and decrease proportions of total motifs before and after the threshold filtering. *N* is the number of total 6mA events, *m* is the number of single motif and *M* is the number of all motifs in each strain.

(4) $$P_{1}=\frac{m}{N}$$

(5) $$P_{2}=\frac{m_{\mathrm{peak}}}{N_{\mathrm{peak}}}$$

(6) $$P_{3}=\frac{m\left(i\geq \mathrm{thres}\right)}{N\left(i\geq \mathrm{thres}\right)}$$

(7) $$I=\frac{M\left(i\geq \mathrm{thres}\right)}{N\left(i\geq \mathrm{thres}\right)}-\frac{M}{N}$$

(8) $$P_{4}=\frac{m_{\mathrm{peak}}\left(i\geq \mathrm{thres}\right)}{N_{\mathrm{peak}}\left(i\geq \mathrm{thres}\right)}$$

(9) $$D=\left(1-\frac{M}{N}\right)-\frac{N\left(i\geq \mathrm{thres}\right)-M\left(i\geq \mathrm{thres}\right)}{N}$$

## Results

### Influence of the Thresholds

**FIGURE 2:**
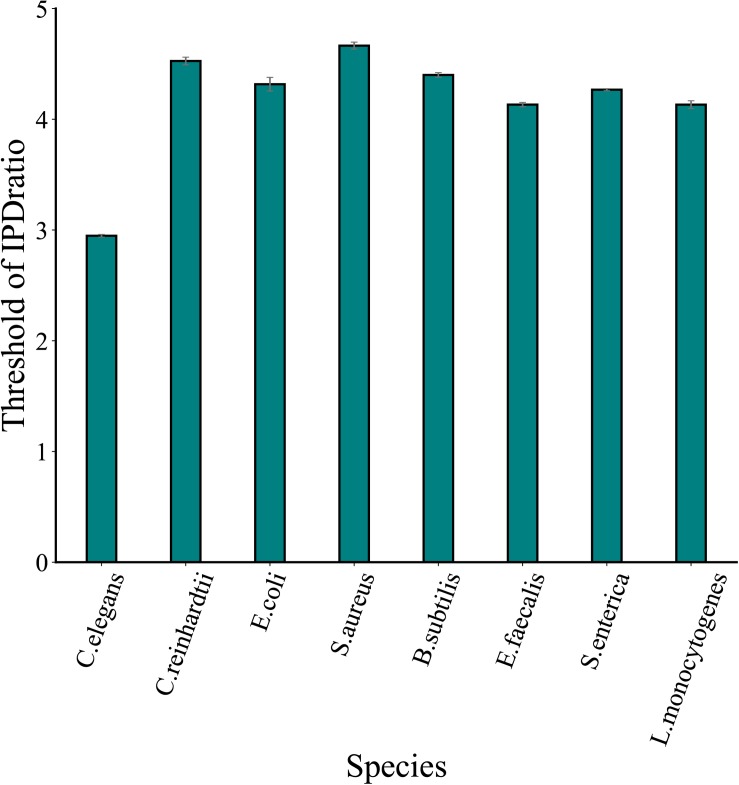
The thresholds of IPD ratio across eight species datasets. The thresholds are generated by MASQC three times tests for eight species.

The proposed method MASQC sets the lower bound of the confidence interval which infers from the IPD ratio of the sample as the threshold. However, it must be point out that the threshold would change for different experiments resulting from the sampling bias. To assess the stability of the thresholds generated by MASQC, we tested the datasets of eight species three times. As shown in Figure 2, the deviations of three thresholds in each species are very small, the result indicates that thresholds bias generated by MASQC have little effect on the final results after filtration (Supplementary Table 1).

### Comparative Analysis of Single Motif

**FIGURE 3:**
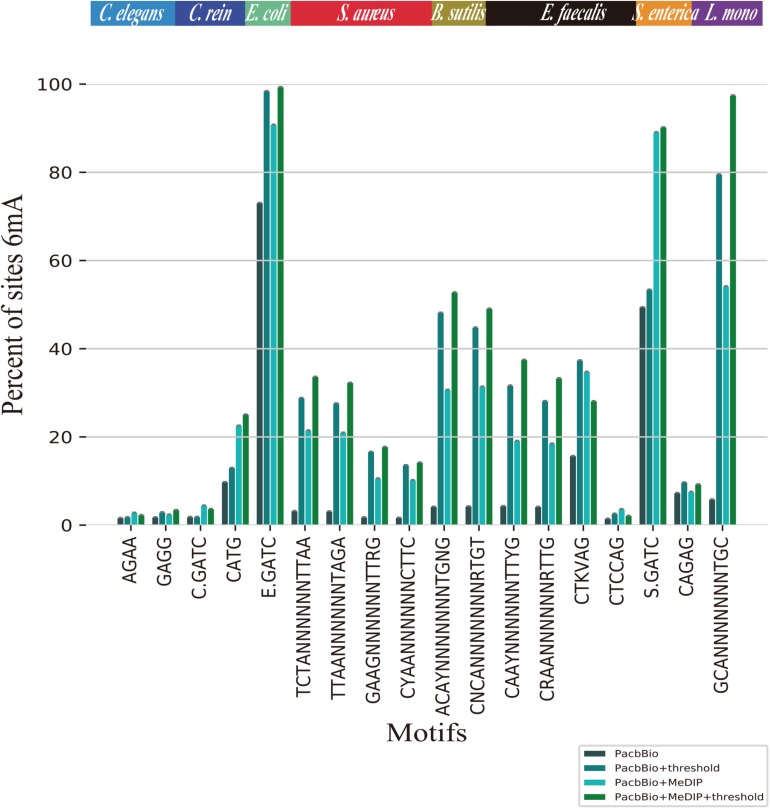
Proportions of identified 6mA-containg motifs for *C. elegans*, *C. reinhardtii*, *E. coli*, *S. aureus*, *B. subtilis*, *E. faecalis*, *S. enterica*, *L. monocytogenes*. The sites 6mA at different single motifs are identified as methylated by PacBio, PacBio + threshold, PacBio + MeDIP, PacBio + MeDIP + threshold across eight species.

To compare the proportions of 6mA-containing motifs per species before and after filtration, we selected 18 motifs from two eukaryotic and six bacterial genomes (McIntyre et al., 2019). AAGANNNNNCTC and GAGNNNNNTCTT in *E. coli*, GATCGVNY in *S. aureus*, BATGCATV in *S. enterica* and ANARAGTANYR in *L. monocytogenes* are with small size, resulting in a lower probability of containing 6mA events. As shown in Figure 3, the proportions of 6mA-containg motifs in threshold filtered *C. elegans, C. reinhardtii, E. coli, S. aureus, B. subtilis, E. faecalis, S. enterica, L. monocytogenes* were significantly higher than that without threshold filtering, but in prokaryotes, the proportions of 6mA-containg motifs in peak regions before and after filtering were stable. The result suggests that the threshold can filter out a large number of non-motifs events and few motifs which may contain true 6mA events. As for *C. elegans and C. reinhardtii*, thresholds filtration did not significantly increase the proportions of 6mA-containg motifs, which was related to the fact that 6mA events in eukaryotes were weakly motif driven.

### Comparative Analysis of Filtered 6mA Events

**FIGURE 4:**
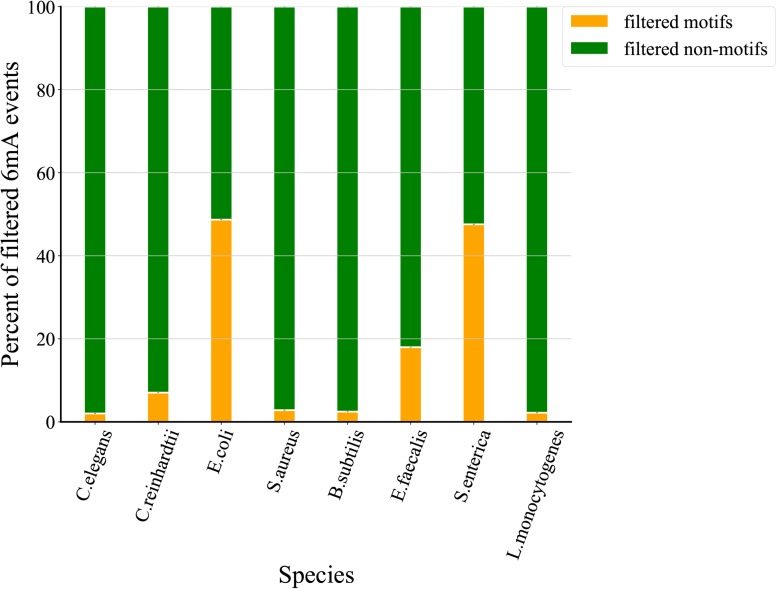
Comparison of proportions of filtered motifs and non-motifs. The green indicates the proportion of filtered motifs and the orange indicates the proportion of filtered non-motifs sites.

To assess the quality of 6mA events filtered through MASQC, we compared the motif and non-motif proportions of IPD ratio below threshold for all 6mA events. As shown in Figure 4. Recent studies identified that the events on the motifs are most likely to be 6mA events than those on the non-motif. The filtered out non-motif events proportions are 98.0, 93.0, 51.3, 97.2, 97.5, 82.0, 52.4, 97.8% for *C. elegans*, *C. reinhardtii*, *E. coli*, *S. aureus*, *B. subtilis*, *E. faecalis*, *S. enterica*, *L. monocytogenes*, which are higher than those of filtered out motifs. The above conclusions suggest that most of the 6mA events filtered out by the proposed threshold may be false positive.

### Comparative Analysis of Total Motifs in Each Species

**FIGURE 5:**
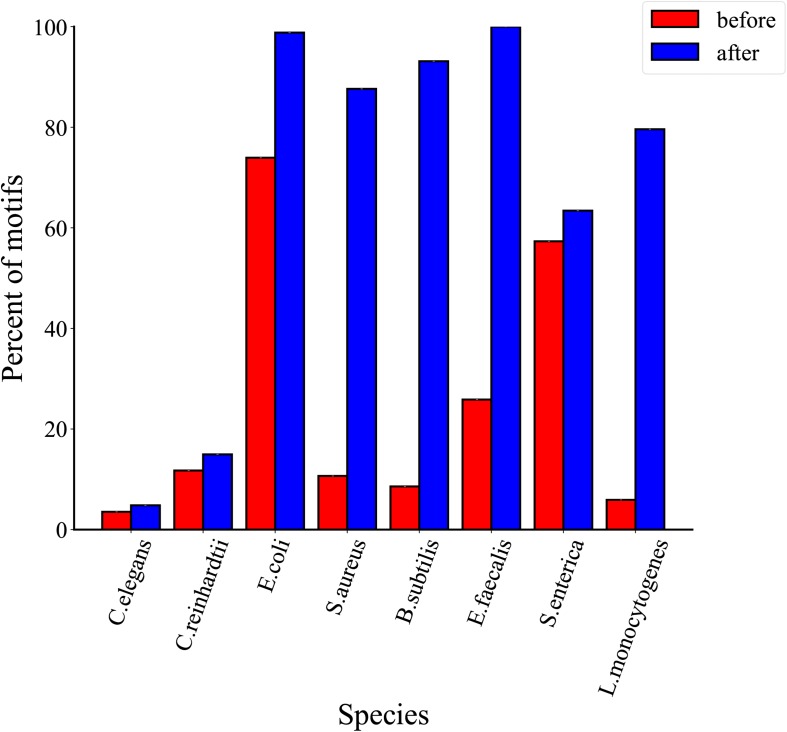
Comparison of proportions of total motifs before and after three threshold filtrations for eight species.

In order to analyze the distribution of total motifs, we compared their proportions before and after threshold filtration. The proportions of total motifs are represented in Figure 5, we found that the proportions of the total motifs increase slightly after three thresholds filtrations for *C. elegans* and *C. reinhardtii*. As the 6mA events in eukaryotes are motif driven weakly and the proportions of 6mA events on motifs are <3%, a growth of 1.3% for *C. elegans* and 3.2% for *Chlamydomonas* after thresholds filtration. On the contrary, 6mA methylation is motif driven highly in bacteria and the proportions of 6mA events on motifs are >95%, so that the proportions of the total motifs are greatly improved compared with eukaryotes. In detail, there is a growth of 24.8% for *E. coli*, 76.9% for *S. aureus*, 84.5% for *B. subtilis*, 74.1% for *E. faecalis*, 6.1% for *S. enterica*, and 73.7% for *L. monocytogenes*. The above results indicate that the proportions of total motifs increase after threshold filtrations in both eukaryotes and prokaryotes.

### Comparative Analysis of Non-motifs Events in Each Species

**FIGURE 6:**
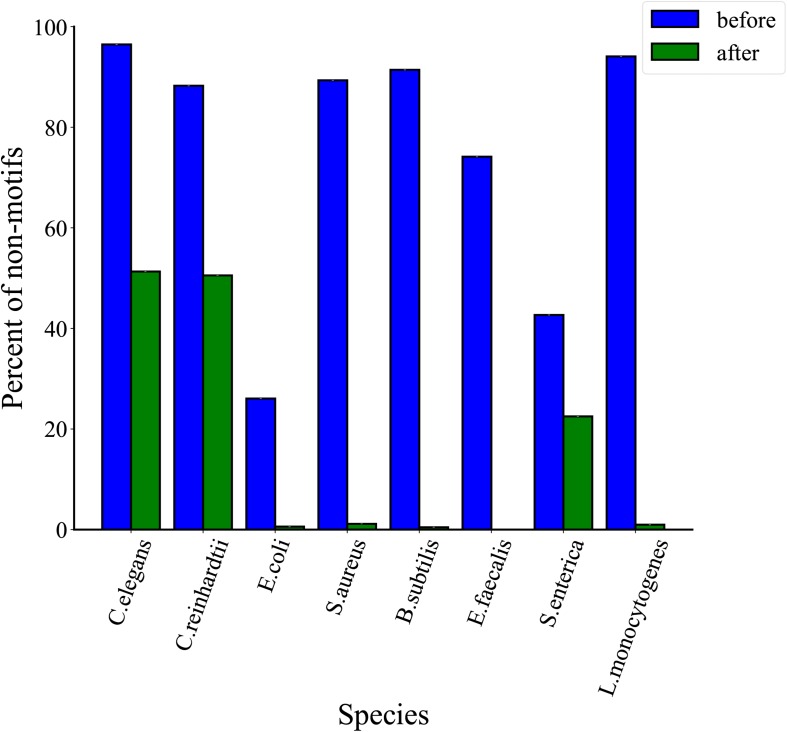
Comparison of proportions of non-motifs sites before and after three threshold filtrations for eight species.

In order to determine the effectiveness of the proposed method, we further analyzed the distribution of non-motifs events before and after thresholds filtration. As shown in Figure 6, the proportions of non-motifs events decrease after three thresholds filtrations in eight species. In detail, there is a decrease of 45.2% for *C. elegans*, 37.7% for *C. reinhardtii*, 25.5% for *E. coli*, 88.2% for *S. aureus*, 90.9% for *B. subtilis*, 74.1% for *E. faecalis*, 20.2% for *S. enterica* and 93.1% for *L. monocytogenes*. A comparative analysis of Figures 5, 6 shows that the proposed MASQC can effectively filter out many fake 6mA events on non-motifs and few fake 6mA events on motifs.

### DNA N6-Methyladenine Identification in *C. reinhardtii*

*Chlamydomonas* is a kind of classic eukaryotic model organism. Fu et al. identified the 6mA modification in 84% of genes in *Chlamydomona*s through MeDIP-seq, enzyme-treated DNA-seq, MNase-seq and RNA-seq (Fu et al., 2015). Fang used WGA and Pacbio SMRT sequencing to detect 6mA in *C. reinhardtii* at a single base level for the first time, which improved the accuracy of the 6mA identification and reduced false positives in the eukaryotic (Zhu et al., 2018). Similarly, we made use of the dataset of *C. reinhardtii* to assess the proposed method MASQC.

**FIGURE 7:**
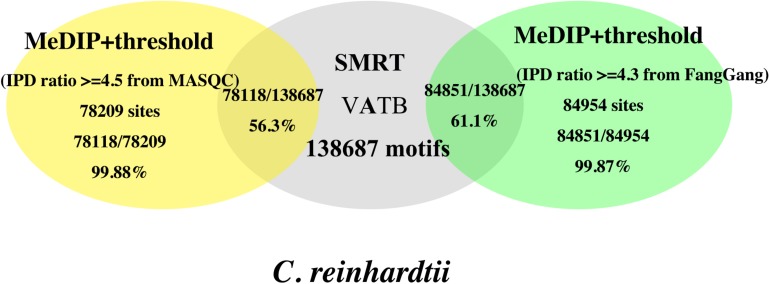
Comparison of accuracies of the 6mA events and VATB (V = A/G/C, B = G/C/T) motifs in *C. reinhardtii* using the threshold from MASQC and Fang’s method. The yellow ellipse is the proportion of the 6mA sites and motifs filtered by MASQC; the gray ellipse is the number of total motifs in *C. reinhardtii*; The green ellipse is the proportion of the 6mA sites and motifs filtered by Fang’s method.

We got the IPD ratio ≥4.3 by applying Fang’s method in our data, which achieved 99.87% accuracy in *C. reinhardtii* motifs, although it achieved better performance, the whole genome SMRT sequencing cost a lot and required the WGA sequencing data as a control (Zhu et al., 2018). Herein, we calculated the threshold of IPD ratio by MASQC and then the 6mA events can be filtered by threshold directly. As shown in Figure 7, the accuracies of threshold from MASQC and Fang’s methods to identify 6mA events and motifs in *C. reinhardtii* were compared.

The threshold derived from the proposed method MASQC is about 4.5. When we used IPD ratio ≥4.5 to filter the 6mA events in peak regions, 99.88% motifs out of the filtered 6mA events and 56.3% VATB motifs out of all VATB motifs (yellow ellipse) in *C. reinhardtii*. The results filtered by IPD ratio ≥4.3 are 99.87% motifs out of the filtered 6mA events and 61.1% VATB motifs out of all VATB motifs (green ellipse) in *C. reinhardtii.* The comparison indicates that our method’s performance is as good as Fang’s method, and our method needs not do WGA sequencing, which saves the cost of the sequencing.

## Discussion

DNA N6-methyladenine (6mA) mainly exists in prokaryotic genomes (Ratel et al., 2006). Recently, 6mA has been discovered in eukaryotic genome, which opened up a new and promising direction for epigenetics research. With the development of specific antibodies and high-throughput sequencing technologies in the past 3 years, 6mA modification has made great breakthroughs in the research of different species. For PacBio SMRT-seq, base modification would affect DNA polymerase kinetics, and then could express different IPD. SMRT-seq can detect not only 6mA events specifically, but also any forms of DNA modifications of DNA polymerase kinetics that is significantly affected by IPD (Michael et al., 2018). Different types of DNA modification (DNA damage, m5C, and derivatives produced during demethylation) at or adjacent to the sites of interest may produce an IPD ratio similar to that of the adenine site, resulting in a high FPR of 6mA events (Flusberg et al., 2010). In bacterial genomes, DNA methylation is relatively limited in form (6mA, 5mC, 4mC) (Yu et al., 2015) and highly motif driven, which greatly reduces the difficulty of detecting and distinguishing 6mA events from other DNA modifications. In contrast, the 6mA events in the eukaryotic genome are much more abundant and motif is driven weakly, that is why it may coexist with other forms of DNA modifications. These differences between eukaryotic and bacterial methylation groups require to be noted when interpreting a hypothetical 6mA call based on SMRT sequencing to avoid misinterpreting false positive events.

This work aims to develop a common computational method to control the quality of 6mA events identification from SMRT sequencing in both eukaryotic and prokaryotic genomes. Fang et al. proposed a method to identify 6mA methylation events in eukaryotes based on both native DNA and whole genome amplification of the same sample without 6mA methylations (Zhu et al., 2018). Although it had an accurate performance of about 80% in Fang’s paper, the whole genome SMRT sequencing is extremely expensive. In this paper, the proposed MASQC controls the FPR of 6mA events with the help of MeDIP-seq datasets. With the help of peak regions from MeDIP-seq datasets, we filtered the 6mA events detected by SMRT sequencing and calculated the threshold of IPD ratio directly to filter out a large number of false positive events. The results indicate that the accuracy of the proposed MASQC could be up to about 99.88% in *C. reinhardtii* which is as good as 99.87% by Fang’s method.

It is worth to note that the 6mA sites filtered by the proposed MASQC may contain a small number of false 6mA events, but they have little effect on the further study of subsequent epigenetics. Researchers can use both parameters “fraction > 0.7” and threshold generated by MASQC to perform more rigorous filtration and get a more conservative truly 6mA dataset.

## Data Availability Statement

All datasets generated for this study are included in the article/Supplementary Material. Scripts used for analysis and figure generation are available at https://github.com/yang-siqian/MASQC.

## Author Contributions

QD and YC conceived and designed the project. SY implemented the algorithms and provided theoretical analysis of the algorithms. SY and YW analyzed the data. QD, SY, and YW wrote the manuscript.

## Conflict of Interest

The authors declare that the research was conducted in the absence of any commercial or financial relationships that could be construed as a potential conflict of interest.

## Footnotes

1 https://www.pacb.com/products-and-services/analyticalsoftware/smrt-analysis/
