# Supplementary material for: MASQC: Next Generation Sequencing Assists Third Generation Sequencing for Quality Control in N6-Methyladenine DNA Identification
Source: Front Genet. 2020 Mar 24;11:269. doi: 10.3389/fgene.2020.00269 (PMC7109398; doi:10.3389/fgene.2020.00269)
Supplement: Supplementary file 1 [file Data_Sheet_1.PDF]

Supplementary Material

1 Supplementary Tables

Supplementary Table 1. Predicted m6A motifs sites predicted by PacBio across eight species. The table lists the reported 6ma-containing motifs, the proposed thresholds of IPDratio, total 6mA sites of each motif before filtered, total 6mA sites of each motif in peaks before filtered, 6mA sites of each motif after filtered and 6mA sites of each motif in peaks after filtered.

Species	Motif	Threshold	Total sites before filtered	Sites in peaks before	Total sites after filtered	Sites in peaks after
C.elegans(3)	G**A**GG	2.9	4849	12	4173	9
	AG**A**A		4399	14	2633	6
C.reinhardtii(3)	G**A**TC	4.5	3151	3039	1394	1357
	C**A**TG		16207	15295	11155	10813
E.coli(4)	G**A**TC	4.3	39043	24071	26470	16300
	AAG**A**NNNNNCTC		207	98	52	19
	G**A**GNNNNNTCTT		219	98	40	13
B.subtills(3)	AC**A**YNNNNNNNTGNG	4.4	522	521	400	400
	CNC**A**NNNNNNNRTGT		534	533	372	372
E.faecalis(5)	CA**A**YNNNNNNTTYG	4.1	864	809	599	571
	CRA**A**NNNNNNRTTG		832	783	533	507
	CTKV**A**G		3100	1469	707	428
	CTCC**A**G		298	155	50	33
S.aureus(6)	TCT**A**NNNNNNTTAA	4.6	408	398	334	324
	TT**A**ANNNNNNTAGA		399	389	320	311
	GA**A**GNNNNNNTTRG		230	197	193	171
	CYA**A**NNNNNNCTTC		218	190	158	137
	G**A**TCGVNY		79	8	8	1
S.enterica(4)	G**A**TC	4.2	23514	15002	15625	10000
	CAG**A**G		3497	1279	2853	1033
	BATGC**A**TV		223	99	60	32
L.monocytogenes(3)	GC**A**NNNNNNNTGC	4.1	869	851	562	549
	ANARAGTANYR		3	0	0	0

Supplementary Table 2. Software and Algorithms. The table lists the softwares and Algorithms Source used in this article.

Software and Algorithms	version	Source
PacBio SMRT analysis	2.3.0	https://www.pacb.com/products-and-services/analytical-software/smrt-analysis/analysis-applications/epigenetics/
BWA	\	http://maq.sourceforge.net
MACS	2	http://liulab.dfci.harvard.edu/MACS/
R	3.5	
Python	3.6	

Supplementary Table 3. SMRT-seq and MeDIP-seq raw data source for eight species. The table lists the SMRT-seq and MeDIP-seq raw data source used in this paper. The columns show the specise names, genome reference for read alignments, Source of raw data, and SRA accession numbers, respectively.

Species	Reference	SMRT and MeDIP-seq anddata Source
C.elegans	ce10	SAMN03699785<br>SAMN03699784
C.reinhardtii	Chlamydomonas_reinhardtii_v5.5	SAMN06639227<br>SAMN06649853<br>SAMN03567036<br>SAMN03567039<br>SAMN03567037
E.coli	ecoli_pb.fasta	SAMN10365290
B.subtills	bsubtills_pb.fasta	SAMN09475310
E.faecalis	efaecalis_pb.fasta	SAMN09475313
S.aureus	saureus_pb.fasta	SAMN09475317
S.enterica	senterica_pb.fasta	SAMN09475319
L.monocytogenes	lmonocytogenes_pb.fasta	SAMN09475315

Supplementary Table 4. Thresholds for three tests from MASQC.

(Supplement_table_4.xlsx)

Supplementary Table 5. Percent of 6mA-containg motifs for eight species by PacBio,PacBio+threshold, PacBio+MeDIP, PacBio+MeDIP+threshold.

(Supplement_table_5.xlsx)

Supplementary Table 6. Percent of motifs before and after filteration.

(Supplement_table_6.xlsx)

Supplementary Table 7. Percent of non-motifs before and after filteration.

(Supplement_table_7.xlsx)
